# Hemodynamic Heterogeneity of Reduced Cardiac Reserve Unmasked by Volumetric Exercise Echocardiography

**DOI:** 10.3390/jcm10132906

**Published:** 2021-06-29

**Authors:** Tonino Bombardini, Angela Zagatina, Quirino Ciampi, Rosina Arbucci, Pablo Martin Merlo, Diego M. Lowenstein Haber, Doralisa Morrone, Antonello D’Andrea, Ana Djordjevic-Dikic, Branko Beleslin, Milorad Tesic, Nikola Boskovic, Vojislav Giga, José Luis de Castro e Silva Pretto, Clarissa Borguezan Daros, Miguel Amor, Hugo Mosto, Michael Salamè, Ines Monte, Rodolfo Citro, Iana Simova, Martina Samardjieva, Karina Wierzbowska-Drabik, Jaroslaw D. Kasprzak, Nicola Gaibazzi, Lauro Cortigiani, Maria Chiara Scali, Mauro Pepi, Francesco Antonini-Canterin, Marco A. R. Torres, Michele De Nes, Miodrag Ostojic, Clara Carpeggiani, Tamara Kovačević-Preradović, Jorge Lowenstein, Adelaide M. Arruda-Olson, Patricia A. Pellikka, Eugenio Picano, on behalf of the Stress Echo 2020 Study Group of the Italian Society of Cardiovascular Imaging

**Affiliations:** 1 Clinical Center of The Republic of Srpska, Faculty of Medicine, University of Banja-Luka, 78000 Banja-Luka, Bosnia and Herzegovina; tbombardini@yahoo.it (T.B.); mostojic2011@gmail.com (M.O.); tamara.kovacevic@medicolaser.info (T.K.-P.); 2 Cardiology Department, Saint Petersburg University Clinic, Saint Petersburg University, 199034 St Petersburg, Russia; zag_angel@yahoo.com; 3 Cardiology Division, Fatebenefratelli Hospital, 82100 Benevento, Italy; 4 Cardiodiagnosticos, Investigaciones Medicas, C1082 ACB Buenos Aires, Argentina; rosinaarbucci@hotmail.com (R.A.); pablommerlo@gmail.com (P.M.M.); lowediego@hotmail.com (D.M.L.H.); lowensteinjorge@hotmail.com (J.L.); 5 Cardiothoracic Department, University of Pisa, 56100 Pisa, Italy; doralisamorrone@gmail.com; 6 Department of Cardiology-Umberto I° Hospital Nocera Inferiore (Salerno)-L. Vanvitelli University of Campania, 84014 Nocera Inferiore, Italy; antonellodandrea@libero.it; 7 Cardiology Clinic, Clinical Center of Serbia, Medical School, University of Belgrade, 11000 Belgrade, Serbia; skali.ana7@gmail.com (A.D.-D.); branko.beleslin@gmail.com (B.B.); misa.tesic@gmail.com (M.T.); belkan87@gmail.com (N.B.); voja2011@yahoo.com (V.G.); 8 Hospital Sao Vicente de Paulo e Hospital de Cidade, 99010-080 Passo Fundo, Brazil; jlpretto@cardiol.br; 9 Cardiology Division, Hospital San José, 88801-250 Criciuma, Brazil; clarissabdaros@cardiol.br; 10 Cardiology Department, Ramos Mejia Hospital, C1221 ADC Buenos Aires, Argentina; miguelamor68@gmail.com (M.A.); hmosto@gmail.com (H.M.); michael.f.salame@gmail.com (M.S.); 11 Cardio-Thorax-Vascular Department, Echocardiography Lab, Policlinico Vittorio Emanuele, Catania University, 95124 Catania, Italy; inemonte@gmail.com; 12 Cardio-Thoracic-Vascular-Department, University Hospital “San Giovanni di Dio e Ruggi d’Aragona”, 84125 Salerno, Italy; rodolfocitro@gmail.com; 13 Heart and Brain Center of Excellence, University Hospital, 5800 Sofia, Bulgaria; ianasimova@gmail.com (I.S.); martina_vl@abv.bg (M.S.); 14 Department of Cardiology, Bieganski Hospital, Medical University, 93-487 Lodz, Poland; wierzbowska@ptkardio.pl (K.W.-D.); kasprzak@ptkardio.pl (J.D.K.); 15 Cardiology Department, Parma University Hospital, 43100 Parma, Italy; ngaibazzi@gmail.com; 16 Cardiology Department, San Luca Hospital, 55100 Lucca, Italy; lacortig@tin.it; 17 Nottola Cardiology Division, 53045 Siena, Italy; chiara_scali@yahoo.it; 18 Centro Cardiologico Monzino, IRCCS, 20138 Milano, Italy; Mauro.Pepi@cardiologicomonzino.it; 19 Highly Specialized Rehabilitation Hospital Motta di Livenza, Cardiac Prevention and Rehabilitation Unit, 31045 Treviso, Italy; antonini.canterin@gmail.com; 20 Department of Cardiology, Federal University of Rio Grande do Sul, 90040-060 Porto Alegre, Brazil; mtorres.mt10@gmail.com; 21 Biomedicine Department, CNR, Institute of Clinical Physiology, 56124 Pisa, Italy; denesm@ifc.cnr.it (M.D.N.); claracarpeggiani@gmail.com (C.C.); picano@ifc.cnr.it (E.P.); 22 Department of Cardiovascular Diseases, Mayo Clinic, Rochester, MN 55901, USA; ArrudaOlson.Adelaide@mayo.edu (A.M.A.-O.); pellikka.patricia@mayo.edu (P.A.P.)

**Keywords:** cardiac reserve, end-diastolic volume, end-systolic volume, heart rate, stress echocardiography

## Abstract

Background: Two-dimensional volumetric exercise stress echocardiography (ESE) provides an integrated view of left ventricular (LV) preload reserve through end-diastolic volume (EDV) and LV contractile reserve (LVCR) through end-systolic volume (ESV) changes. Purpose: To assess the dependence of cardiac reserve upon LVCR, EDV, and heart rate (HR) during ESE. Methods: We prospectively performed semi-supine bicycle or treadmill ESE in 1344 patients (age 59.8 ± 11.4 years; ejection fraction = 63 ± 8%) referred for known or suspected coronary artery disease. All patients had negative ESE by wall motion criteria. EDV and ESV were measured by biplane Simpson rule with 2-dimensional echocardiography. Cardiac index reserve was identified by peak-rest value. LVCR was the stress-rest ratio of force (systolic blood pressure by cuff sphygmomanometer/ESV, abnormal values ≤2.0). Preload reserve was defined by an increase in EDV. Cardiac index was calculated as stroke volume index * HR (by EKG). HR reserve (stress/rest ratio) <1.85 identified chronotropic incompetence. Results: Of the 1344 patients, 448 were in the lowest tertile of cardiac index reserve with stress. Of them, 303 (67.6%) achieved HR reserve <1.85; 252 (56.3%) had an abnormal LVCR and 341 (76.1%) a reduction of preload reserve, with 446 patients (99.6%) showing ≥1 abnormality. At binary logistic regression analysis, reduced preload reserve (odds ratio [OR]: 5.610; 95% confidence intervals [CI]: 4.025 to 7.821), chronotropic incompetence (OR: 3.923, 95% CI: 2.915 to 5.279), and abnormal LVCR (OR: 1.579; 95% CI: 1.105 to 2.259) were independently associated with lowest tertile of cardiac index reserve at peak stress. Conclusions: Heart rate assessment and volumetric echocardiography during ESE identify the heterogeneity of hemodynamic phenotypes of impaired chronotropic, preload or LVCR underlying a reduced cardiac reserve.

## 1. Introduction

The goal of the heart during exercise is to increase cardiac output (CO) to metabolizing tissues [1]. Cardiac reserve is defined as an appropriate increase in CO during stress and requires adequate contractile, preload and chronotropic reserves [1]. Stress-induced myocardial ischemia and regional wall motion abnormalities may cause reduced cardiac reserve, but cardiac reserve can be impaired also in the absence of inducible ischemia. During treadmill or bicycle exercise, heart rate (HR) normally increases two- to three-fold, left ventricular contractile reserve (LVCR) three- to four-fold, and systolic blood pressure by ≥50%, while systemic vascular resistance decreases. LV end-diastolic volume (EDV) initially increases for the rise in venous return to sustain the augmentation in stroke volume (SV) through the Frank–Starling mechanism and later decreases at high HR. CO during mild exercise is achieved by an augmentation of both SV and HR, whereas the further increase in output during intense exercise results primarily from an increase in HR [2,3]. Two-dimensional volumetric exercise stress echocardiography (ESE) provides an integrated view of preload reserve through EDV and LVCR through end-systolic volume (ESV) changes. The current study hypothesis is that volumetric ESE can identify patients with abnormal reserve of cardiac index (CI, i.e., CO/body surface area), and the underlying hemodynamic phenotype due to lack of EDV increase or inadequate reduction in ESV including the force-based assessment of LVCR as the stress/rest ratio of force, calculated as systolic blood pressure/end-systolic volume [3,4]. The simultaneous EKG assesses chronotropic response. The identification of such heterogeneous hemodynamic phenotypes is a prerequisite for an effective and personalized therapeutic approach. We therefore analyzed the CI, HR, SV, EDV, ESV and LVCR data in 1344 patients with known or suspected coronary artery disease referred to ESE without stress-induced regional wall motion abnormalities recruited in the Stress Echo 2020 study [4]. 

## 2. Materials and Methods

### 2.1. Study Population

In this prospective study, we evaluated 1344 patients (550 female; 794 male; age 59.8 ± 11.4 years; left ventricular ejection fraction (EF) 63 ± 8%, mean ± SD) recruited from 1 September 2016 to 1 September 2018 by 21 laboratories in eight countries (Argentina, Brazil, Bulgaria, Hungary, Italy, Poland, Russian Federation, Serbia) [4]. The inclusion criteria were: (1) Age > 18 years; (2) referral to ESE for known or suspected coronary artery disease (CAD) with normal (≥50%, *n* = 1241) or near normal (40–49%, *n* = 103) resting LV EF [5]; (3) no severe primary valvular or congenital heart disease, without severe mitral insufficiency at peak stress; (4) wall motion imaging of acceptable quality at rest, with adequate visualization of at least 16 out of 17 segments in at least one view, and contrast enhancement when ≥2 segments were inadequately visualized; (5) sinus rhythm; (6) negative stress echo (peak ≤ rest wall motion score index); (7) willingness to give their written informed consent allowing scientific utilization of observational data. Of the initial population of 1,924 patients initially enrolled, 552 (28.6%) were excluded because of premature termination of the exercise test for inducible ischemia at stress; 28 for arrhythmias (i.e., 20 persistent atrial fibrillation; eight paced rhythm). The remaining 1344 (69.9%) were included in the final analysis. All patients underwent ESE testing as part of a clinically-driven evaluation according to the referring physician’s indications. The study protocol was reviewed and approved by the institutional ethics committees as a part of the SE 2020 study (148-Comitato Etico Lazio-1, 16 July 2016; Clinical trials. Gov Identifier NCT 030.49995). The study was funded partly by the Italian National Research Council (Ageing project) and with travel grants of the Italian Society of Cardiovascular Imaging with dedicated sessions during national meetings. No support from industry was received. 

### 2.2. Exercise Testing Procedures

All exercise tests were performed on a semi-supine bicycle ergometer (*n* = 1217, 90.6%) or a motorized treadmill using the Bruce protocol (*n* = 127, 9.4%). Exercise testing procedures outlined by the American Heart Association were followed for all assessments [6]. All patients were continuously monitored with 12-lead EKG, and hemodynamic measurements were made during each stage of the protocol. Blood pressure was measured with an automated sphygmomanometer with auditory confirmation. Patients were encouraged to exercise to their maximum tolerance. Percentage of age-predicted maximal HR was determined by dividing peak HR for maximal age-predicted HR (220-age) multiplied by 100 [6]. Maximal rate-pressure-product was defined as the product of the highest HR and systolic blood pressure obtained during the last stage of exercise [6].

### 2.3. Stress Echocardiography

We used commercially available ultrasound machines. All patients underwent comprehensive transthoracic echocardiography at rest. Patients underwent ESE with semi-supine (25 watts increments every 2 min) or post-treadmill exercise, according to the recommended protocols [7]. Electrocardiogram and blood pressure were monitored continuously. The imaging protocol of SE was used when each laboratory had completed the upstream quality control process [8]. Echocardiographic imaging was performed from parasternal long axis view, short axis view, and apical 4-, 3- and 2-chamber view, using conventional 2-dimensional echocardiography. Wall motion score index was calculated in each patient at baseline and peak stress, in a four-point score ranging from 1 (normal) to 4 (dyskinetic) in a 17-segment model of the left ventricle [8]. The force-based assessment of LVCR as the stress/rest ratio of force, was calculated as systolic blood pressure/end-systolic volume [9]. All doctors and nurses involved in the stress echocardiogram procedures were trained in Basic Life Support and Advanced Cardiac Life Support. The procedure for data acquisition and analysis was standardized through a web-based learning module before starting data collection. All readers (one for each center) underwent a quality control as previously described for assessment of regional wall motion abnormalities [8] and ESV [10].

### 2.4. Volume Analysis

Left ventricular (LV) EDV and ESV were measured from apical four- and two-chamber views, using the biplane Simpson method. When the biplane apical views were not available, a single plane 4-chamber view area-length method was used [7]. Only representative cycles with optimal endocardial visualization were measured and the average of three measurements was taken. The endocardial border was traced, excluding the papillary muscles. The frame captured at the R wave of the EKG was considered to be the end-diastolic frame, and the frame with the smallest left ventricular silhouette, the end-systolic frame. Images were obtained in the same position (semi-supine or upright) for each patient at baseline at peak stress, or immediately after stress in post-treadmill imaging. Cuff systolic blood pressure was recorded at the time of volume measurements both at rest and peak stress. The same readers (one from each center) accredited for regional wall motion assessment also underwent quality control for ESV assessment as detailed elsewhere [10,11]. The quality control of ESV implied reading of a different set of 20 videoclips selected from seven different laboratories. The accepted threshold was ≥90% concordance with area measurement (from apical 4- and 2- chamber views). The gold standard was the average reading of two experienced observers of the coordinating centers. For each clip, the measurement was considered concordant when the reading was ±20% from the gold standard. All cardiac volumes were normalized to body surface area, yielding their respective indexes: EDV index; ESV index and SV index. Preload reserve impairment was defined as LV EDV stress < LVEDV rest.

### 2.5. Heart Rate Response

Heart rate reserve (HRR) was calculated as the peak/rest HR ratio from 12-lead EKG [12]. Chronotropic incompetence was defined as peak/rest HR increase <1.85.

### 2.6. Stroke Volume Index and Cardiac Index

LV volumes were evaluated by the biplane Simpson and area-length methods. The SV (mL) was calculated as EDV-ESV [13]. The CO (mL/min) was computed as the product of HR and SV as previously reported [14]. All CO and SV were normalized to body surface area, yielding their respective indices: CI and SV index. To assess the dependence of CI upon LVCR and EDV changes and HR during ESE, CI reserve was identified by peak-rest value [15]. Reserve (∆) was defined as the difference in these variables between peak stress and rest.

### 2.7. Data Storage

Results of each test were entered in the digital data registry at the time of testing by each recruiting center and sent monthly to the coordinating center with the electronic case report form including clinical data. After checking for internal consistency by trained technical staff, and double-checking with the center for data verification on possibly inconsistent input, the data were added to the data registry.

### 2.8. Statistical Analysis

Statistical analyses included descriptive statistics (frequency and percentage of categorical variables, and mean and standard deviation of continuous variables). The Pearson chi-square test with Fisher’s exact test for categorical variables and the Mann-Whitney test for continuous variables for intergroup comparisons were performed to confirm significance. Based on CI reserve from rest to peak exercise stress, study patients were assigned into low, medium and high tertiles. One-way analysis of variance was used to analyze differences between hemodynamic parameters at each stage of exercise between three groups. When homogeneity of variance was not present, the Kruskal-Wallis test for nonparametric independent samples was used. An intergroup comparison was performed with Scheffé and Tamhane post hoc tests, respectively. Multivariable factors associated with reduced (lowest tertile) CI reserve during ESE, expressed as a binary variable, were investigated using a Binary Logistic regression model. The initial set of clinical covariates selected included age, sex, diabetes, hypertension, resting EF and wall motion score index. The stress covariates included: chronotropic incompetence, abnormal contractile reserve, reduced preload reserve as categorical variables and peak LV EF, as continuous variable. All variables were included in the model without any transformation. The significant variables at the *p* < 0.1 level in these initial models, were simultaneously entered in a summary binary logistic regression model. Statistical significance was set at *p* < 0.05. Statistical Package for the Social Sciences ver. 22.0 (SPSS Inc., Chicago, IL, USA) was adopted for analysis.

## 3. Results

Patients in the middle and highest tertiles were younger, more often male, with less diabetes, hypertension, history of dyspnea and previous myocardial infarction, and lower prevalence of beta-blocker therapy (Table 1). 

Highest tertiles compared with the lowest tertile had higher SV index reserve, higher peak HR, and greater increase of EDV index at peak stress (Table 2).

### 3.1. Hemodynamic Correlates of Normal Cardiac Index Reserve

Patients in the middle and highest tertile of CI reserve (Group 1) showed one or more of the following abnormalities: altered HRR in 327 patients (36.5%); altered preload reserve (with peak EDV index < resting EDV index) in 467 patients (52.1%); altered LVCR in 455 patients (50.8%) (Table 2); 451 patients (50.3%) showed one abnormality, 332 (37.1%) two abnormalities, 44 (4.9%) three abnormalities, and 69 none (7.7%). 

### 3.2. Hemodynamic Correlates of Abnormal Cardiac Index Reserve

Patients in the lowest tertile of CI reserve (Group 2) showed one or more of the following abnormalities: altered HRR in 303 patients (67.6%, *p* < 0.001 vs. Group 1); altered preload reserve in 341 patients (76.1%, *p* < 0.001 vs. Group 1) with peak EDV index < resting EDV index; altered LVCR in 252 patients (56.3%, *p* = 0.058 vs. Group 1) (Table 2); 108 patients (24.1%) showed one abnormality, 225 (50.2%) two abnormalities, 113 (25.2%) three abnormalities, and two none (0.4%, *p* < 0.001 vs. Group 1). In the overall group of 1344 study patients 559 patients (41.6%) showed one abnormality, 557 (41.4%) two abnormalities, 157 (11.7%) three abnormalities, and 71 none (5.3%). 

### 3.3. Cardiac Index Reserve in Different Hemodynamic Subsets

**Figure 1:**
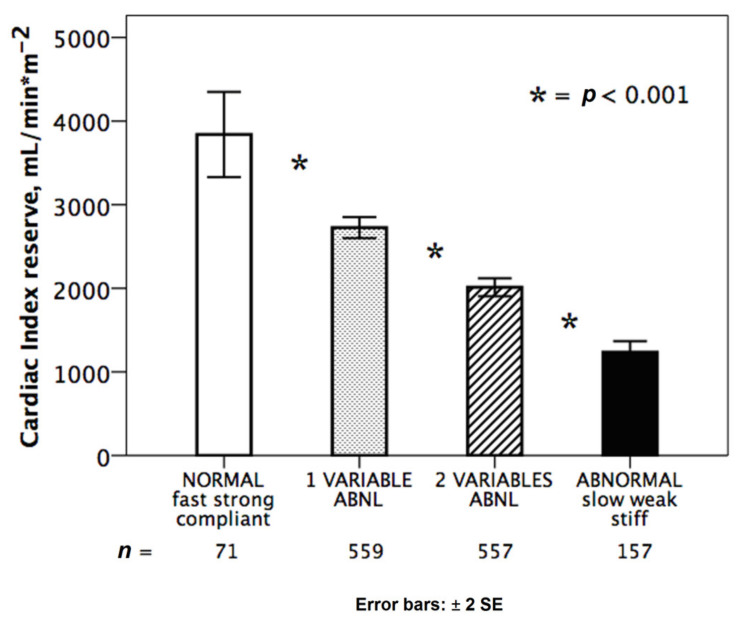
Normal and abnormal hemodynamic responses during stress. Cardiac index reserve in normal response (white bar, *n* = 71): fully abnormal hearts (black bar, *n* = 157) with reduced chronotropic, preload and contractile reserve; partially abnormal responses with one (dotted bar: stiff, *n* = 322; weak, *n* = 170; slow, *n* = 66) or two abnormalities (dashed bar: weak + slow, *n* = 229; stiff + slow, *n* = 178; stiff + weak, *n* = 151). * = *p* < 0.001 across various subsets.

**Figure 2:**
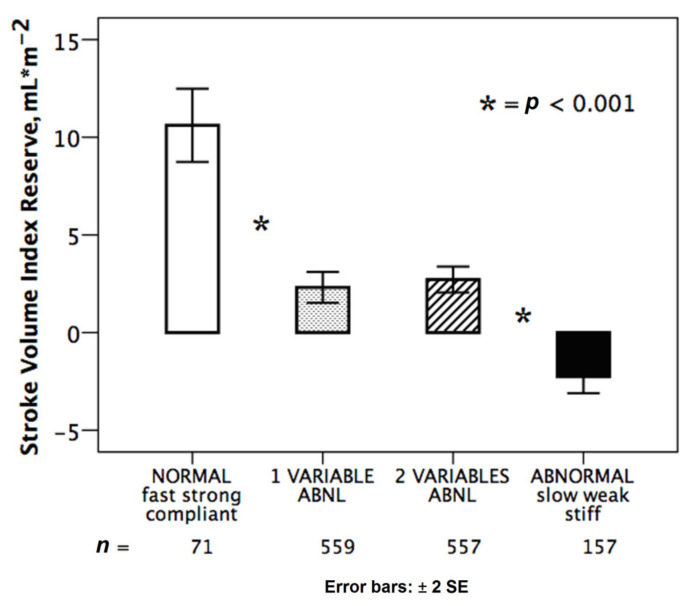
Normal and abnormal hemodynamic responses during stress. Stroke volume index reserve in normal response (white bar): abnormal responses (black bar) with reduced chronotropic, preload and contractile reserve; partially abnormal responses with one (dotted bar: stiff, *n* = 322; weak, *n* = 170; slow, *n* = 66) or two abnormalities (dashed bar: weak + slow, *n* = 229; stiff + slow, *n* = 178; stiff + weak, *n* = 151). * = *p* < 0.001 across various subsets.

The integration of HRR, preload reserve and LVCR allowed to identify different groups clustered on the basis of the hemodynamic pattern for CI reserve (Figure 1) and for SV index reserve (Figure 2). 

We identified four main subgroups: normal heart (with preserved chronotropic, preload and contractile reserve); abnormal heart (with impaired chronotropic preload and contractile reserve); a group with two abnormalities and a group with 1 abnormality. At binary logistic regression analysis, independent variables associated with the lowest tertile of CI reserve at peak stress were the following: the reduced preload reserve with an odds ratio of 5.6; chronotropic incompetence with an odds ratio of 3.9; and reduced LVCR with an odds ratio of 1.6 (Table 3).

## 4. Discussion

A multi-parametric approach instead of a single parameter approach can be used to exploit at the fullest the unique versatility of ESE. Patients with a normal cardiac reserve show a preserved chronotropic, preload and contractile reserve, but patients with abnormal cardiac reserve have heterogeneous alterations in chronotropic, preload or contractile reserve (Table 2). This information can be obtained without additional software, without extra-imaging time, and with very limited extra-analysis time required to measure volumes off-line [15]. Lower HR increase, EDV decrease at stress, and abnormal LVCR were associated with an increased likelihood of reduced cardiac reserve (Table 3). Hypertension and diabetes were associated with higher probability of reduced cardiac reserve, suggesting that in absence of signs of regional dysfunction an early global hemodynamic impairment can be detected in these conditions during stress.

### 4.1. Comparison with Previous Studies 

Previous studies have shown the possibility and usefulness of extracting information on chronotropic incompetence, preload reserve and contractile reserve from EKG and 2-dimensional echocardiography to characterize the hemodynamic response of the heart under stress. HRR is an imaging-independent parameter that helps to identify a subset with reduced sympathetic reserve and worse outcome, as shown in the pre-stress imaging era by Ellestadt et al. [16] and Lauer et al. [17], and with ESE by Elhendy et al. [18]. During SE, this information has been shown to be independent of stress-induced regional wall motion abnormalities in predicting prognosis [7,18]. The assessment of chronotropic incompetence can be a cause of dyspnea in heart failure with preserved EF (HFpEF) and has been incorporated in the 2017 recommendations for SE applications beyond CAD [19], since its presence is a specific and treatable cause of inadequate CI during stress. In our population of patients referred for CAD, we found a reduced chronotropic reserve in 630 (46.9%) patients, suggesting that this mechanism is effective in a significant portion of patients referred to SE. These findings are consistent with a recent meta-analysis including 910 patients showing that the most frequent and severe hemodynamic abnormality observed in patients with HFpEF was the impairment of chronotropic reserve [20]. A second cause for inadequate CI reserve is an abnormal LVCR. The SE approach to assessing LVCR is reasonably simple and accurate, and we found it in 252 (56.3%) of patients with a reduced CI reserve. This is in line with what has been found by Kasner et al. [21] in a population of 52 patients referred for HFpEF. They found a blunted LVCR in 25 (48%) of patients with symptoms during exercise. Interestingly, we observed that 455/707 (64%) of patients with reduced LVCR had a preserved CI reserve (merged medium-high tertiles), possibly due to a preserved preload reserve with capability to increase EDV during stress (Figure 2). The third cause for inadequate CI reserve is a stiff heart, with inability to increase EDV at comparable HR. Again, this is consistent with what has been found by Shimiaie et al. [22] who observed a blunted increase in LV EDV in 16 patients with HFpEF and dyspnea on effort. EDV was comparable to healthy controls at rest, but significantly lower at initial steps of unloaded effort, at anaerobic threshold and at maximal effort. Several studies have shown that LV diastolic alterations are more manifest during exercise than at rest. This results from progressive volume unloading of the LV due to limited relaxation reserve in combination with increased LV passive stiffness, despite preserved force–frequency relation, i.e., normal LVCR [23].

These three mechanisms have been described in the past, assessed with varying techniques and in different populations. The novelty of our approach is that they can now converge conceptually, logistically, and methodologically in the 2-dimensional volumetric ESE combined by simple EKG and exploiting information already present in the SE minimum image and data set. This allows to partially overcome the well-known limitations of EF, which has gained widespread acceptance due to its ease of application and wealth of clinical data supporting its use, yet is highly dependent on loading conditions and gives no information on the hemodynamic mechanisms underlying the development of heart failure and a reduced cardiac reserve. The proposed approach uses clinically available information (EDV, ESV, HR) for identification of different phenotypes of exercise response which can be theoretically be targeted by SE-driven tailored therapeutic intervention.

### 4.2. Clinical Implications 

**Figure 3:**
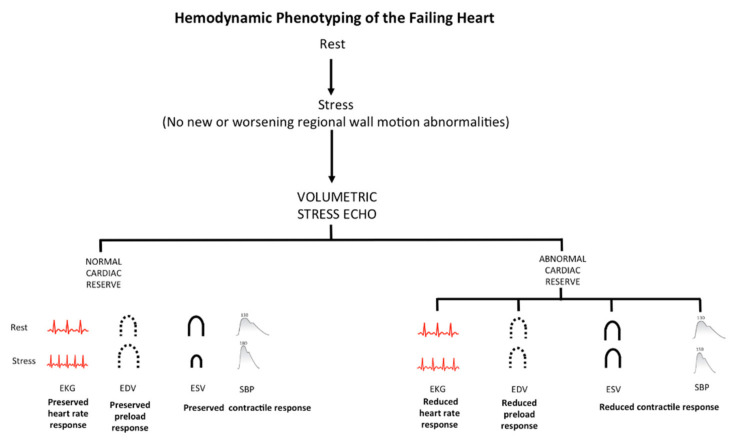
Hemodynamic phenotyping of the failing heart by stress echocardiography. SE based on regional wall motion abnormalities easily allow to identify inducible myocardial ischemia as regional wall motion abnormalities often associated with abnormal cardiac reserve (upper panel). However, a normal response of regional wall motion can also be associated with abnormal cardiac reserve, possibly due to the isolated or combined presence of distinct phenotypes: reduced heart rate response with chronotropic incompetence; “reduced preload response” with impaired preload reserve; “reduced contractile response” with blunted LVCR. Each phenotype must be identified for effective personalized treatment.

The current recommended protocol of ESE includes evaluation of HR and EF, derived from EDV and ESV. These same data can be used for a more comprehensive reconstruction of the cardiovascular adaptation to exercise including HRR, LVCR and preload reserve. Due to missing evidence, this information finds no place in current recommendations for diastolic stress echo, which are based only on estimation of E/e’ and systolic pulmonary artery pressure through tricuspid regurgitant jet velocity [19]. Yet, the comprehensive evaluation of chronotropic, contractile and preload reserves provide a unique and appealingly simple approach to detect specific hemodynamic patterns in cardiovascular abnormalities (Figure 3).

This hemodynamic characterization during stress might pave the way to personalized treatment of heart failure by a novel phenotype-driven therapy. Insufficient cardio-acceleration because of reduced HRR could be a therapeutic target with rate-adaptive pacing [24] or beta-blocker deprescribing [25]. Abnormal preload reserve might suggest cautious use of diuretics in presence of signs of pulmonary congestion such as rest or stress B-lines with lung ultrasound [26]. Cardiac contractility modulation therapy might be more beneficial in patients with an impaired LVCR [27]. 

### 4.3. Limitations

We measured left ventricular volumes with 2-dimensional echocardiography, which is highly feasible and accurate, especially when rest-stress variations are assessed in the same patient [7,11,13]. It requires geometrical assumptions and the risk of possible foreshortening of the left ventricle during stress, but images needed for the analysis are the same as those utilized for regional wall motion analysis. There is potential room for further methodological improvement with operator-independent cardiac volumetric analysis with 3-dimensional transthoracic echocardiography using an automated adaptive analytics algorithm [28]. However, it is important to prove today, with available technologies present in every laboratory, the feasibility and potential of the method- which may only increase when coupled with new technologies and advanced imaging [7]. The separate analysis of the chronotropic, preload and contractile reserves is conceptually and practically helpful, but there is obvious interaction between HR, preload and contractility. The increase in HR also increases contractility through the well-known Bowditch-Treppe effect [11]. Tachycardia reduces EDV as higher HR reduces duration of diastole much more than systole. When the duration of the cardiac cycle decreases significantly from baseline (cardiac cycle = 1000 msec at 60 bpm; cardiac cycle = 333 msec at 180 bpm) the longer duration of diastolic time at rest is reduced to equalize the systolic time component (≈50% and 50%) in the normal heart. The relative decrease in diastole time does not allow sufficient diastolic filling time with the inability of the heart to receive venous return, and a pulmonary and systemic venous engorgement occurs [29]. However, patients with preload reserve reduction had comparable peak HR in our series, suggesting that a genuine abnormality of preload reserve can be detected even at maximal HR. We evaluated only exercise, the most physiological and used stress, with echocardiography, the simpler and more affordable imaging method. However, the novel approach described herein can be applied to all imaging techniques assessing volumes such as stress cardiovascular magnetic resonance and to all stresses, although the reference values for volume and CO normal response are obviously stress-specific, being higher with the stronger chronotropic and inotropic stress such as dobutamine compared to vasodilators such as dipyridamole [7]. The phenotypic approach was focused on the three main variables underlying a reduced cardiac reserve, which outlined the heterogeneous basis of the same defect (an abnormal cardiac reserve). This is probably a step forward compared to the standard approach, and uses an information which is already present in the minimum data set. The multiparametric approach allowed by ESE has much more to offer in unselected populations, since the same abnormal cardiac reserve can be due for instance to diastolic dysfunction or dynamic LV obstruction, all possibly converging in an abnormality in cardiac reserve. For these reasons, the approach now adopted in the Stress Echo 2030 study (the Evolution of Stress Echo 2020) is more comprehensive, with assessment also of global strain for step C (cardiac, contractile and preload reserve of left ventricle), and systematic evaluation of step F (mitral insufficiency), step G (dynamic gradients), step L (left atrial volume), step P (pulmonary and LV pressures) and step R (right ventricular function) when needed in several patients’ subsets. Stroke volume calculation by the volumetric method (EDV-ESV) will be compared with the Doppler data. We pooled data from semi-supine (>90%) and treadmill ESE, reflecting the variability of ESE practice in the real world. Both tests are recommended in guidelines and show similar volumetric changes in non-ischemic hearts with EDV increase (possibly smaller with treadmill) and ESV decrease [7]. Sensitivity analysis to recognize abnormal cardiac reserve (i.e., low cardiac index reserve, first tertile) in the whole group of 1344 patients was 67.63% for reduced heart rate response, 76.12% for reduced preload response and 56.25% for reduced contractile response with a negative predictive value respectively of 79.69%, 80.04%, and 69.23%. Excluding treadmill ESE patients, sensitivity analysis to recognize abnormal cardiac reserve (i.e., low cardiac index reserve, first tertile) in the group of 1217 semi-supine ESE was 67.40% for reduced heart rate response, 79.41% for reduced preload response and 54.17% for reduced contractile response with a negative predictive value respectively of 79.88%, 80.73%, and 69.09%.

## 5. Conclusions

Volumetric ESE can be used to identify and quantify preload and contractile reserve during exercise, while the simultaneous EKG assesses the chronotropic reserve. An impairment of cardiac reserve is associated with impairment of one or more of these three contractile, preload and chronotropic reserves. The detection of the heterogeneity of hemodynamic phenotypes underlying the final common pathway of a reduced cardiac reserve is the prerequisite for a targeted and personalized treatment with a phenotype-guided approach [30]. 

## Figures and Tables

**Table 1:** Study patients and classes of cardiac reserve.

	Overall (*n* = 1344)	Low CI Reserve First Tertile (*n* = 448)	Medium CI Reserve Second Tertile (*n* = 448)	High CI Reserve High Tertile (*n* = 448)	*p* Value
Male gender, *n* (%)	794 (59.1%)	244 (54.5%)	254 (56.7%)	296 (66.1%)	0.001
Age (years)	59.8 ± 11.4	62.6 ± 11.1	60.4 ± 10.4	56.4 ± 11.8	<0.001
Hypertension, *n* (%)	909 (67.6%)	333 (74.3%)	321 (71.7%)	255 (56.9%)	<0.001
Diabetes mellitus, *n* (%)	249 (18.5%)	107 (23.9%)	84 (18.8%)	58 (12.9%)	<0.001
History of dyspnea, *n* (%)	129 (9.6%)	54 (12.1%)	48 (10.7%)	27 (6%)	0.006
History of myocardial infarction, *n* (%)	344 (25.6%)	137 (30.6%)	106 (23.7%)	101 (22.5%)	0.012
History of PCI/CABG, *n* (%)	327 (24.3%)	145 (32.4%)	102 (22.8%)	80 (17.9%)	<0.001
Beta blockers, *n* (%)	601 (44.7%)	248 (55.4%)	207 (46.2%)	146 (32.6%)	<0.001
Nitrates, *n* (%)	47 (3.5%)	27 (6%)	14 (3.1%)	6 (1.3%)	0.001
Calcium channel blockers, *n* (%)	211 (15.7%)	86 (19.2%)	73 (16.3%)	52 (11.6%)	0.007
Statins, *n* (%)	648 (48.2%)	229 (51.1%)	233 (52%)	186 (41.5%)	0.002
ACEi/ARB, *n* (%)	727 (54.1%)	260 (58%)	259 (57.8%)	208 (46.4%)	<0.001
Diuretics, *n* (%)	43 (3.2%)	7(1.6%)	18 (4%)	18 (4%)	0.055
Anti-platelet agents, *n* (%)	719 (53.5%)	265 (59.2%)	257 (57.4%)	197 (44%)	<0.001

Clinical parameters according to subdivision in CI tertiles: the first CI reserve tertile (≤1591 mL/min × m^−2^ CI increase), the second CI tertile (>1591 mL/min × m^−2^ and ≤2599 mL/min × m^−2^ CI increase) and the highest CI reserve tertile (CI increase >2599 mL/min × m^−2^). Data are presented as mean value with SD of continuous variables; frequency and percentage of categorical variables. ACEi/ARB, angiotensin-converting enzyme inhibitors/angiotensin receptor blockers; BSA, body surface area; CABG, coronary artery bypass graft; PCI, percutaneous coronary intervention.

**Table 2:** Hemodynamic, rest and stress echo findings according to CI reserve.

	Overall (*n* = 1344)	Low CI Reserve First Tertile (*n* = 448)	Medium CI Reserve Second Tertile (*n* = 448)	High CI Reserve High Tertile (*n* = 448)	*p* Value
Heart rate, beats/min					
Rest	71.2 ± 12.1	72.3 ± 13.5	71.6 ± 11.5	69.8 ± 11.1	0.007 ^
Peak	132.8 ± 19.2	122.9 ± 20.0	133.3 ± 15.9	142.1 ± 16.3	<0.001 *
Peak/rest	1.90 ± 0.35	1.73 ± 0.30	1.90 ± 0.31	2.07 ± 0.35	<0.001 *
Mean pressure, mmHg					
Rest	94.0 ± 11.5	93.3 ± 12.3	94.8 ± 10.6	94.1 ± 11.5	0.143
Peak	121.1 ± 16.3	118.4 ± 17.8	121.7 ± 15.8	123.4 ± 14.8	<0.001°
Reserve	27.1 ± 15.7	25.1 ± 17.5	26.9 ± 14.7	29.3 ± 14.4	<0.001 ^
End-diastolic volume index, mL × m^−2^					
Rest	52.0 ± 21.7	44.6 ± 16.0	46.9 ± 15.5	64.4 ± 26.2	<0.001 ^
Peak	48.8 ± 20.2	37.1 ± 12.4	44.7 ± 12.2	64.6 ± 22.8	<0.001 *
Reserve	−3.1 ± 11.2	−7.4 ± 10.5	−2.2 ± 8.9	0.2 ± 12.4	<0.001 *
End-systolic volume index, mL × m^−2^					
Rest	20.0 ± 10.9	16.8 ± 7.8	17.4 ± 7.6	25.7 ± 13.8	<0.001 ^
Peak	14.4 ± 9.0	12.6 ± 7.4	12.9 ± 7.0	17.8 ± 11.1	<0.001 ^
Reserve	−5.3 ± 6.3	−4.2 ± 5.2	−4.4 ± 4.7	−7.9 ± 7.7	<0.001 ^
Stroke volume index, mL × m^−2^					
Rest	32.0 ± 12.9	27.8 ± 10.1	29.6 ± 9.6	38.7 ± 15.3	<0.001 ^
Peak	34.4 ± 14.5	24.6 ± 7.4	31.8 ± 7.3	46.8 ± 16.2	<0.001 *
Reserve	2.4 ± 8.6	−3.2 ± 6.9	2.2 ± 5.7	8.1 ± 8.8	<0.001 *
Ejection fraction (%)					
Rest	62.5 ± 8.0	62.9 ± 8.0	63.4 ± 7.3	61.2 ± 8.3	<0.001°
Peak	71.2 ± 10.2	67.8 ± 10.8	72.2 ± 8.7	73.7 ± 10.1	<0.001°
Reserve	8.7 ± 7.6	4.9 ± 7.8	8.8 ± 6.0	12.5 ± 6.9	<0.001 *
Left ventricular contractile reserve (LVCR, Peak/Rest Force)					
Rest Force (mmHg/mL)	4.17 ± 1.99	4.83 ± 2.28	4.45 ± 1.71	3.23 ± 1.53	<0.001 *
Peak Force (mmHg/mL)	9.38 ± 6.33	10.70 ± 7.65	9.52 ± 5.19	7.94 ± 5.63	<0.001 *
LVCR	2.32 ± 1.33	2.29 ± 1.61	2.17 ± 0.93	2.51 ± 1.35	0.001 ^
WMSI (ratio)					
Rest	1.110 ± 0.265	1.110 ± 0.232	1.066 ± 0.190	1.152 ± 0.343	<0.001 §
Peak	1.064 ± 0.179	1.086 ± 0.200	1.046 ± 0.153	1.061 ± 0.179	0.003 ^
Viability, *n* (%)	157 (11.7%)	47 (10.5%)	42 (9.4%)	68 (15.2%)	0.016
Cardiac index, mL/min × m^−2^					
Rest	2276 ± 991	2013 ± 834	2119 ± 818	2695 ± 2271	<0.001 ^
Peak	4590 ± 2125	2980 ± 904	4194 ± 897	6596 ± 2271	<0.001 *
Reserve	2314 ± 1507	967 ± 515	2075 ± 288	3901 ± 1441	<0.001 *
Stress impaired hemodynamic phenotypes, *n* (%)					
Reduced heart rate response	630 (46.9%)	303 (67.6%)	214 (47.8%)	113 (25.2%)	<0.001 *
Reduced preload response	808 (60.1%)	341 (76.1%)	252 (56.3%)	215 (48.0%)	<0.001 *
End-systolic volume increase	222 (16.5%)	95 (21.2%)	66 (14.7%)	61 (13.6%)	0.004 ^
Reduced contractile response	707 (52.6%)	252 (56.3%)	247 (55.1%)	208 (46.4%)	0.006 ^

The first CI reserve tertile (≤1591 mL/min × m^−2^ CI increase), the second CI tertile (>1591 mL/min × m^−2^ and ≤2599 mL/min × m^−2^ CI increase) and the highest CI reserve tertile (CI increase >2599 mL/min × m^−2^). Data are presented as mean value with SD of continuous variables, frequency and percentage of categorical variables. Significance between tertiles: * all; ^ High tertile vs. medium and low; ° Low tertile vs. medium and high; § Medium tertile vs. high and low. CI, Cardiac index; LVCR, left ventricular contractile reserve; WMSI, wall motion score index.

**Table 3:** Univariable and multivariable binary logistic regression analysis of factor associated with abnormal. Cardiac index reserve.

	Univariable Analysis		Multivariable Model	
OVERALL (*n* = 1344)				
Variables	OR (95%CI)	*p*	OR (95%CI)	*p*
Female Sex	1.329 (1.056–1.672)	0.015	1.819 (1.372–2.413)	<0.001
Age	1.035 (1.024–1.046)	<0.001	1.027 (1.013–1.040)	<0.001
Hypertension (yes)	1.609 (1.250–2.070)	<0.001		
Diabetes (yes)	1.666 (1.257–2.208)	<0.001		
Rest WMSI	0.984 (0.642–1.509)	0.942		
Rest Ejection Fraction %	1.009 (0.995–1.024)	0.205		
Peak Ejection Fraction %	0.951 (0.940–0.962)	<0.001	0.932 (0.916–0.948)	<0.001
Inability to perform 85% age-predicted HR	2.752 (2.177–3.749)	<0.001	2.363 (1.177–3.140)	<0.001
Blunted LVCR (peak FORCE/rest FORCE ≤ 2)	1.246 (0.992–1.565)	0.059	1.579 (1.105–2.259)	0.012
Chronotropic incompetence (stress/rest HR < 1.85)	3.636 (2.860–4.623)	<0.001	3.923 (2.915–5.279)	<0.001
Reduced preload reserve (peak EDV < rest EDV)	2.928 (2.272–3.773)	<0.001	5.610 (4.025–7.821)	<0.001

Lower tertile, ≤1591 mL/min × m^−2^ Cardiac index increase. CI, confidence intervals; HR, heart rate; OR, odds ratio; WMSI, wall motion score index.

## Data Availability

The data presented in this study are available on request from the corresponding author. The data are not publicly available due to privacy law.

## Abbreviation

The following abbreviations are used in this manuscript:

CI	cardiac index
CO	cardiac output
EDV	end-diastolic volume
EF	ejection fraction
ESE	exercise stress echocardiography
ESV	end-systolic volume
HR	heart rate
HRR	heart rate reserve
LVCR	left ventricular contractile reserve
SV	stroke volume
